# PCR-based detection of *Enterocytozoon bieneusi* in diarrheic patients from Guangdong, Shandong, Shanghai, and Zhejiang Provinces, China: a study on prevalence and genotypic characteristics

**DOI:** 10.3389/fcimb.2025.1728500

**Published:** 2026-01-09

**Authors:** Jiangqiong Ke, Lijie Sun, Qi Yu, Xiaorong Yu, Zhongkai Zhang, Aiying Jiang, Xin Peng, Jiabin Zhang, Fuhao Jiang, Yanyan Jiang, Huanhuan Zhou, Wei Zhao

**Affiliations:** 1 Department of Geriatrics, The Second Affiliated Hospital and Yuying Children’s Hospital of Wenzhou Medical University, Wenzhou, Zhejiang, China; 2 Department of Clinical Laboratory, The Fifth Affiliated Hospital Sun Yat-sen University, Zhuhai, China; 3 Department of Clinical Laboratory, Shanghai Pudong New Area People’s Hospital, Shanghai, China; 4 Laboratory Department, Qingdao South District Center for Disease Control and Prevention, Qingdao, Shandong, China; 5 School of Basic Medical Sciences, Wenzhou Medical University, Wenzhou, China; 6 National Institute of Parasitic Diseases, Chinese Center for Disease Control and Prevention (Chinese Center for Tropical Diseases Research); NHC Key Laboratory of Parasite and Vector Biology, Shanghai, China

**Keywords:** China, diarrhea, *Enterocytozoon bieneusi*, genotyping, human

## Abstract

**Introduction:**

*Enterocytozoon bieneusi* is the most prevalent microsporidian species infecting humans and causing diarrhea. Epidemiological investigations seldom focus on this pathogen, and its disease burden has been underestimated. This research investigated the prevalence and genotypic characteristics of *E. bieneusi* in patients with diarrhea from Guangdong (Zhuhai), Shandong (Qingdao), Shanghai and Zhejiang (Wenzhou) Provinces, China.

**Methods:**

A total of 691 fecal specimens from patients exhibiting diarrhea were collected and subjected to polymerase chain reaction (PCR) detection, targeting the internal transcribed spacer (ITS) region of the *E. bieneusi* genome. Genotypes were identified by sequencing PCR products, and zoonotic risk was evaluated through homology and phylogenetic analysis.

**Results:**

4.9% (34/691) of patients were positive for *E. bieneusi*. The patients from Shanghai had the highest incidence at 14.3% (21/147), which was significantly higher than those in Qingdao (2.9%, 5/171), Wenzhou (3.3%, 3/90), and Zhuhai (1.8%, 5/283) (χ^2^ = 35.5, *P* < 0.001). Meanwhile, the positive rate was 7.1% (18/254), 3.1% (10/325) and 5.4% (6/112) among children, adults, and the elderly, respectively. Additionally, the positive rate of patients in rural areas was 8.6% (26/302), significantly higher than that in urban areas, which was 2.1% (8/389) (χ^2^ = 15.6, *P* < 0.001). Twelve genotypes of *E. bieneusi* were identified, including seven known genotypes: CHG3 (n = 17), CHG19 (n = 3), EbPigITS7 (n = 3), Type IV (n = 3), CHG5 (n = 1), EbpA (n = 1), and S7 (n = 1), as well as five novel genotypes (SHH1, WZH1, ZHH1, ZHH2 and ZHH3), each represented by a single sample. The identified genotypes can be categorized into Groups 1, 2, 12, and 13 through phylogenetic analysis.

**Conclusions:**

This study offers insights into the epidemiology and genetic diversity of *E. bieneusi* among patients experiencing diarrhea in four provinces of China. It also underscores the necessity of ongoing monitoring and management to prevent the spread of this pathogen.

## 1 Introduction

*Enterocytozoon bieneusi*, an obligatory intracellular pathogen, is a widely-distributed fungus (Zhang et al., 2021). It has been detected in human populations across 42 countries (Wang et al., 2024). Infected individuals may exhibit symptoms such as persistent diarrhea, abdominal pain, vomiting, fever, malabsorption, and/or weight loss (Nourrisson et al., 2024). The severity of the infection is contingent upon the individual’s immune status. Immunocompetent individuals may experience a less severe, self-limiting *E. bieneusi* infection that typically resolves within weeks or months, and some may be asymptomatic. Nevertheless, *E. bieneusi* acts as an opportunistic pathogen in immunocompromised individuals, including patients with human immunodeficiency virus (HIV)/acquired immunodeficiency syndrome (AIDS) and organ transplant recipients. In these patients, microsporidiosis can give rise to a wasting disease and may even culminate in death (Nourrisson et al., 2024). *E. bieneusi* has also been identified in 170 animal species. A hypothesis suggests that it is a zoonotic pathogen capable of transmission between humans and animals (Zhang et al., 2021). Moreover, its presence in drinking water and its association with food-borne outbreaks have been reported (Hu et al., 2014; Izquierdo et al., 2011; Michlmayr et al., 2022). Transmission occurs through the fecal-oral route; humans become infected by coming into contact with infected animals or individuals, or by ingesting water or food contaminated with infectious spores (Nourrisson et al., 2024). Unfortunately, due to the lack of a vaccine for microsporidiosis and the treatment challenges patients encounter, it is significant to eliminate the infection source and interrupt transmission routes to control microsporidiosis caused by *E. bieneusi* (Nourrisson et al., 2024).

Molecular techniques are employed to identify and trace the transmission routes and sources of *E. bieneusi* infection. Nested polymerase chain reaction (PCR) targeting the internal transcribed spacer (ITS) region is extensively utilized to detect *E. bieneusi* DNA in fecal specimens, thereby defining thousands of genotypes (Santín and Fayer, 2009; Zhang et al., 2021). Among these genotypes, certain ones are specific to either humans or animals, while others are shared, which implies the potential for zoonotic transmission (Li et al., 2019). Apart from analyzing genotype overlaps, phylogenetic analyses hold substantial significance. In these analyses, the named genotypes of *E. bieneusi* can be classified into 15 evolutionary branches, denoted as Groups 1 to 15 (Nourrisson et al., 2024). The genotypes in Group 1 demonstrate a low degree of host specificity and possess broad host ranges, suggesting their potential to cause zoonotic concerns (Li et al., 2019). Genotypes in Group 2 were formerly considered to be adapted to ruminants; however, subsequent findings have identified some in other animals and humans. As a result, Group 2 is now regarded as an emerging zoonotic concern (Nourrisson et al., 2024). Genotypes within Groups 3 to 15 are more host-specific, and current data indicate host adaptation in most genotypes of these groups, suggesting a limited zoonotic potential (Nourrisson et al., 2024). Surveillance of genotypes across diverse host populations is indispensable for comprehending transmission patterns and sources, and surveys of susceptible populations are crucial for controlling outbreaks. Although infected individuals may be asymptomatic, diarrhea is the most prevalent symptom. Therefore, cases of diarrhea should be assessed for *E. bieneusi* infection. Testing for *E. bieneus*i in these cases facilitates the identification of sources, enables the implementation of intervention measures, and improves patients’ quality of life.

Research on *E. bieneusi* in China started relatively late, and the first cases of human infections were reported in 2011 (Zhang et al., 2011). Subsequently, epidemiological investigations conducted across 12 provinces indicated that the human infection rates of *E. bieneusi* ranged from 0.2% to 22.5% (**Table 1**). Despite the progress made in research on *E. bieneusi* in China, the existing surveys, especially those focused on human populations, demonstrate significant shortcomings. Major coastal cities, including Qingdao, Shanghai, Wenzhou, and Zhuhai, have complex population structures and exhibit high population mobility. Furthermore, climatic and environmental factors in these regions, such as warm and humid climates, may contribute to the survival and dissemination of *E. bieneusi* spores and could potentially accelerate the spread of the pathogen. The present study intends to conduct an epidemiological investigation of *E. bieneusi* among patients with diarrhea in four coastal cities, namely Qingdao (Shandong), Shanghai, Wenzhou (Zhejiang), and Zhuhai (Guangdong). The objective is to ascertain the infection rates and prevalent genotypes of *E. bieneusi* in these cities, thereby laying a foundation for formulating targeted prevention and control strategies.

**Table 1:** Infection rates and genotype distribution of *E. bieneusi* in different provinces of China.

Provinces	% (No. positive/No. sampled)	Genotype(s) (n)	References
Chongqing	10.6 (14/132)	D (7); CQH5-11 (1each)	Zang et al., 2021
Chongqing	11.8 (11/93)	PigEBITS5 (3); CC2 (2); CQ-H2 (2); CQ-H1 (1); CQ-H3 (2); CQ-H4 (1)	Ding et al., 2018
Guangxi	6.5 (7/50)	D (4); CM1(2); MEB5(1)	Li et al., 2021
Guangxi	11.6 (33/285)	D (11); Type IV (8); PigEBITS7 (7); EbpC (4); GX25 (1); GX456 (1); GX458 (1)	Liu et al., 2017
Hainan	3.7 (47/1,264)	CHG2 (3); CHG3 (5); CHG5 (10); SHR1 (4); Type IV (2); CM21 (1); EbpA (1); EbpC (1); PigEBITS4 (1); PigEBITS7 (1); HNH-1 to HNH-18 (1each)	Zhang et al., 2022
Heilongjiang	9.9 (58/583)	D (39); EbpC (1); CHN-H1 (4); CHN-H2 (1); CHN-H3 (1); NA (12)	Zhao et al., 2022
Heilongjiang	1.4 (3/222)	D (1); YCHH1 (1); YCHH2 (1)	Zhou et al., 2022
Heilongjiang	7.5 (19/255)	CS-4 (2); EbpC (11); Henan-IV (3); NEC1 to NEC5 (1each)	Yang et al., 2014
Heilongjiang	1.3 (5/381)	D (4); HLJ-CP1 (1)	Zhang et al., 2017
Henan	1.2 (27/2284)	D ( 17); J (2); PigEBITS7 (1); BEB6 (1); CM8 ( 1)	Yu et al., 2019
Henan	5.0 (68/1366)	EbpC (39); D (12); type IV (7); PigEBITS7 (1); Peru8 (1); EbpD (1); Henan-I-V (1each); Peru11 (1); Unknown (1)	Wang et al., 2013b
Hubei	5.0 (1/20)	D (1)	Zang et al., 2021
Hubei	0.2 (1/500)	D (1)	Wang et al., 2017
Jilin	22.5 (9/40)	CHN1 (5); CHN3 (4); CHN4 (3); I (3); J (3); CHN2 (2)	Zhang et al., 2011
Shandong	11.4 (5/44)	D (5)	Zang et al., 2021
Shanghai	13.5 (34/252)	NA (34)	Liu et al., 2014
Shanghai	4.2 (24/573)	Peru11 (6); EbpA (2); SH2 (3); SH1 (1); SH3 (1); SH4 (1); EbpC (1); D (1); SH5-12 (1 each)	Wang et al., 2013a
Shanghai	5.2 (8/155)	D ( 2); EbpC (1); TypeIV (1); Peru11 (1); A (1); EbpD (1); I (1)	Jiang et al., 2023
Xinjiang	5.9 (36/609)	A (3); CHN6 (1); CXJH1 (1); CXJH 2 (1); CXJH 3 (1); D (6); EbpA (3); KB-1 (1); NIA1 (19)	Qi et al., 2020
Yunnan	8.3 (24/289)	Peru6 (21); YN104 (1); YN241 (1); YN249 (1)	Gong et al., 2019
Zhejiang	1.6 (3/185)	BEB6 (1); J (1); I (1)	Ye et al., 2025
Zhejiang	7.2 (35/489)	Type IV (14); D (5); NBH8 (5); NBH7(3); I (1); CHN4 (1); NBH1-6 (1each)	Liu et al., 2023

NA: Genotype not identified, positive only at the small subunit site. ITS genotype unavailable.

## 2 Materials and methods

### 2.1 Ethical approval

The protocol for this study received approval from the Ethics Committees of Wenzhou Medical University (Approval number SCILLSC-2021-01, approved on March 4, 2020). Before sample collection, written informed consent was obtained from each participant or their legal guardians if the participant was a minor. The study’s purpose, procedures, potential risks, and benefits were fully explained, and participation was voluntary. Their personal information was kept confidential and used only for this research. The anonymity and privacy of the participants were strictly protected during the study.

### 2.2 Sample collection

**Figure 1:**
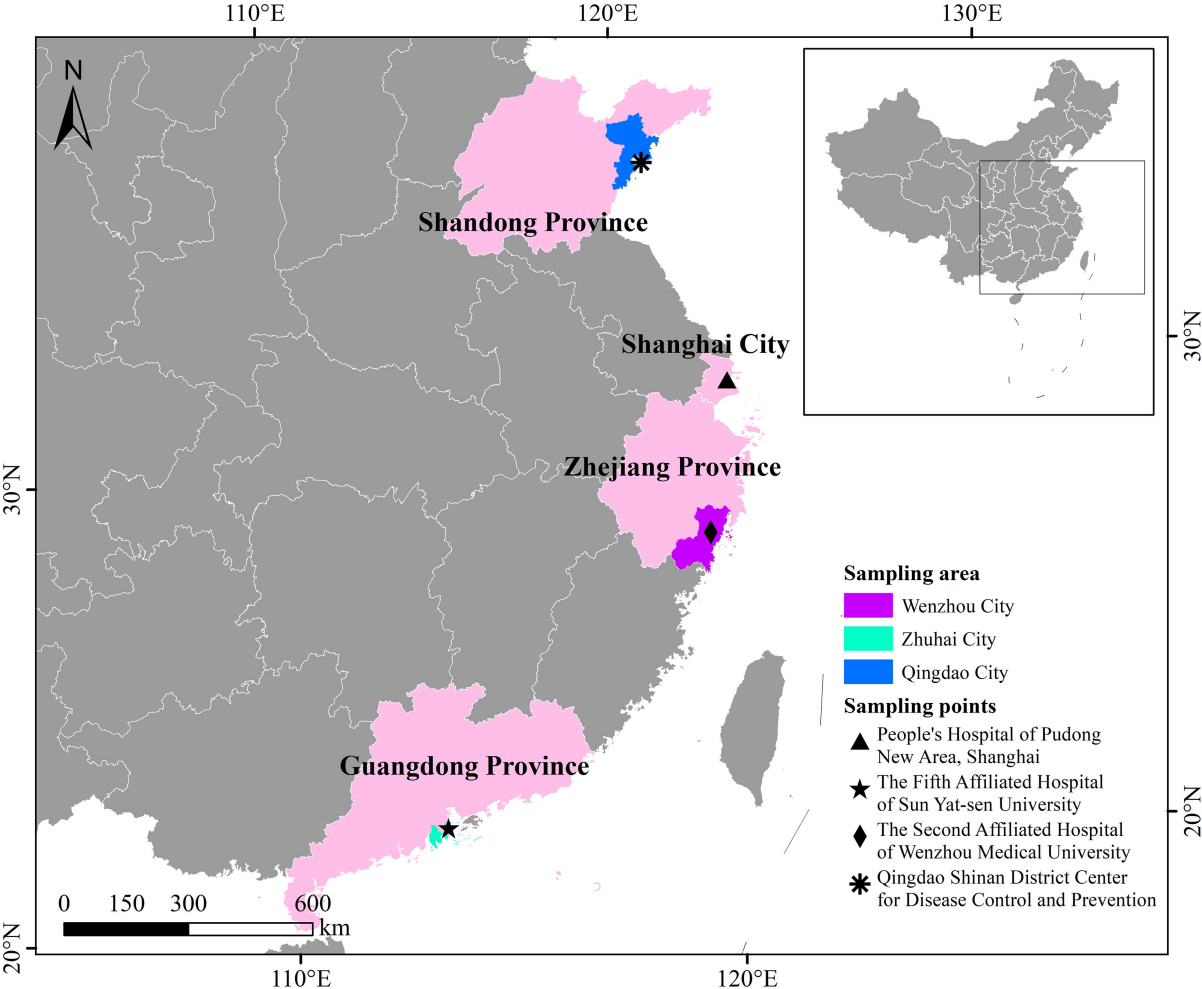
The map shows the geographical locations of hospitals where surveyed diarrhea patients were admitted. The authors first created it with ArcGIS 10.4 software, importing the original vector data from the National Center for Basic Geographic Information (http://www.ngcc.cn). Then, to meet specific copyright and licensing requirements, the final map was edited and overlaid using Microsoft PowerPoint 2003 and Adobe Photoshop CS6.

Between March 2023 and June 2025, a total of 691 fecal specimens were collected from diarrheal patients at the Laboratory Departments of four hospitals located in Shanghai (n = 147), Zhejiang (specifically in Wenzhou, n = 90), Guangdong (in Zhuhai, n = 283), and Shandong (in Qingdao, n = 171) Provinces, China (**Figure 1**). The specimens were obtained from patients who were clinically diagnosed with diarrhea, presenting with fecal excretion exceeding 200 mg and experiencing no fewer than three episodes of diarrhea per day. Prior to sample collection, informed consent was obtained from the patients or from the parents/guardians of minor patients, and they were instructed on the correct method for collecting fecal specimens using a plastic fecal collector. All stool collection containers were labeled with the unique clinical record number (no patient names were included), along with the collection date. The patients’ age, gender, and location (rural or urban) information were extracted from the patients’ medical records by clinicians and properly documented. No other disease information was recorded or statistically analyzed; however, it was confirmed that all these patients were HIV-negative. The collected specimens were subsequently refrigerated at 4 °C and transported via cold chain within 24 hours to the laboratory at Wenzhou Medical University, where they were aliquoted into three 1.5 mL microcentrifuge tubes and stored at -80 °C until DNA extraction.

### 2.3 DNA extraction

DNA was extracted using the QIAamp DNA Stool Mini Kit following the manufacturer’s instructions, with the following modifications: the lysis temperature was increased to 95 °C (Zhao et al., 2014). The eluted DNA was stored at - 20 °C until further utilization in PCR amplification.

### 2.4 PCR amplification

PCR amplification was carried out to detect the presence of *E. bieneusi* in the extracted DNA samples. A nested PCR approach was employed, targeting an approximately 389-base pair (*bp*) fragment, which encompasses 76 bp of the 3’ end of the *SSU rRNA* gene, 243 bp of the internal region, and 70 bp of the 5’ region of the large-subunit *(LSU) rRNA* gene. The primers and PCR cycle settings employed in this PCR assay were previously documented (Buckholt et al., 2002). TaKaRa Taq DNA Polymerase (TaKaRa Bio Inc., Tokyo, Japan) was employed for all PCR amplifications. Both positive (Peru11 DNA from children) and negative (no DNA) controls were included in every PCR assay to guarantee the precision and reproducibility of our findings. The PCR products were then visualized by electrophoresis on a 2% agarose gel stained with GelRed (Biotium Inc., CA, USA). Positive samples, indicated by the presence of a specific band of the expected size, were further processed for sequence analysis to determine the genotype of the *E. bieneusi* present in the samples.

### 2.5 Sequence analyses

The PCR products showing the anticipated band size in agarose gel electrophoresis and the primers used in the nested PCR were directly sent to Sangon Biotech Co., Ltd. (Shanghai, China) for bidirectional sequencing. Intercept 243 bp ITS region from the internal region of the obtained sequence. Subsequently, the Basic Local Alignment Search Tool (BLAST) was utilized to match these sequences with those in the National Center for Biotechnology Information (NCBI). If there were 100% consistency, the sequence was designated as a known genotype and assigned its initial nomenclature; If no 100% identical sequence was found with any single or multiple nucleotide substitutions, deletions, or insertions, and this was confirmed by the DNA sequencing of at least two PCR products, the sequence was considered to be a novel genotype. The naming convention for novel genotypes by combining the initial letters of the sample source city (e.g. SH = Shanghai, ZH = Zhuhai, WZ = Wenzhou, QD = Qingdao) and “human” (H = human), along with Arabic numerals (e.g., 1). The established nomenclature system based on ITS nucleotide sequence was used to determine *E. bieneusi* genotypes.

### 2.6 Phylogenetic analysis

A phylogenetic tree was established using the Neighbor - joining (NJ) approach implemented in the MEGA X software (http://www.megasoftware.net/) (Kumar et al., 2018). The evolutionary distances were calculated by means of the Kimura two - parameter model. The reliability of the tree topology was assessed via bootstrap analysis with 1000 replications.

### 2.7 Statistical analysis

Pearson’s chi-square (χ^2^) was conducted using SPSS version 22.0 (SPSS Inc., Chicago, IL, USA) to respectively analyze the prevalence of *E. bieneusi* across distinct cities, the total genders, residences, and age groups. A *P-*value threshold of less than 0.05 was employed to ascertain statistical significance.

### 2.8 Nucleotide sequence accession numbers

The nucleotide sequences obtained from our current research endeavors have been duly registered in the GenBank database under the following accession numbers: PX583129 to PX583140.

## 3 Results

### 3.1 Prevalence of *E. bieneusi*

An analysis of the ITS region of the *SSU rRNA* gene revealed that *E. bieneusi* was present in 4.9% (34/691) of all samples. The positive rate among patients from Shanghai was the highest at 14.3% (21/147), significantly exceeding rates in the other three cities: Qingdao (2.9%, 5/171), Wenzhou (3.3%, 3/90), and Zhuhai (1.8%, 5/283) (χ^2^ = 35.5; *P* < 0.001). **Table 2** further elaborates on the positive rates of *E. bieneusi* among different genders, age groups, and residential areas in the four cities. In general, the positive rate among males was 4.1% (16/395), lower than the rate among females at 6.1% (18/296); however, this difference was not statistically significant (χ^2^ = 1.49; *P* = 0.22). Children had an positive rate of 7.1% (18/254), which was significantly higher than adults at 3.1% (10/325) (χ^2^ = 4.98; *P* = 0.03), but not significantly different from the rate among the elderly at 5.4% (6/112) (χ^2^ = 0.38; *P* = 0.54). The positive rate in rural areas was 8.6% (26/302), significantly higher than in urban areas at 2.1% (8/389) (χ^2^ = 15.6; *P* < 0.001) (**Table 2**).

**Table 2:** Prevalence and genotype distribution of *E. bieneusi* in diarrheic patients from Shanghai, Qingdao, Wenzhou, and Zhuhai Cities, China.

Groups	Shanghai		Qingdao(Shandong)		Wenzhou (Zhejiang)		Zhuhai (Guangdong)		Total	*P-value*
	Prevalence	Genotype(s) (n)	Prevalence	Genotype(s) (n)	Prevalence	Genotype(s) (n)	Prevalence	Genotype(s) (n)	Prevalence	*P^0^* < 0.001
Gender										*P^1^* = 0.22
Males	12/78 (15.4)	CHG3 (8); CHG19 (2); SHH1 (1); S7 (1)	1/96 (1.0)	EbPigITS7 (1)	2/49 (4.1)	CHG19 (1); WZH1 (1)	1/172 (0.6)	TypeIV (1)	4.1 (16/395)	
Females	9/69 (13.0)	CHG3 (9)	4/75 (5.3)	EbPigITS7 (1); TypeIV (2); EbpA (1)	1/41 (2.4)	CHG5 (1)	4/111 (3.6)	ZHH1 (1); ZHH2 (1); ZHH3 (1); EbPigITS7 (1)	6.1 (18/296)	
Ages										*P^2^ = 0.08*
Children	13/80 (16.3)	CHG3 (9); CHG19 (2); SHH1 (1); S7 (1)	2/70 (2.9)	TypeIV (1); EbpA (1)	–	–	3/104 (2.9)	ZHH1 (1); ZHH2 (1); ZHH3 (1)	7.1 (18/254)	*P^3^ = 0.03*
Adult	6/53 (11.3)	CHG3 (6)	2/70 (2.9)	EbPigITS7 (1); TypeIV (1)	0/39	–	2/163 (1.2)	TypeIV (1); EbPigITS7 (1)	3.1 (10/325)	*P^4^ = 0.54*
Elderly	2/14 (14.3)	CHG3 (2)	1/31 (3.2)	TypeIV (1)	3/51 (5.9)	CHG19 (1); WZH1 (1); CHG5 (1)	0/16		5.4 (6/112)	*P^5^ = 0.27*
Residential location										*P^6^ <0.001*
Rural	20/116 (17.2)	CHG3 (16); CHG19 (2); SHH1 (1); S7(1)	1/31 (3.2)	EbpA (1)	1/32 (3.1)	CHG19 (1)	4/123 (3.3)	ZHH1 (1); ZHH2 (1); ZHH3 (1); TypeIV (1)	8.6 (26/302)	
Urban	1/31 (3.2)	CHG3 (1)	4/140 (2.9)	EbPigITS7 (2); TypeIV (2)	2/58 (3.4)	WZH1 (1); CHG5 (1)	1/160 (0.6)	EbPigITS7 (1)	2.1 (8/389)	
Total	21/147 (14.3)	CHG3 (17); CHG19 (2); SHH1 (1); S7 (1)	5/171 (2.9)	EbPigITS7 (2); TypeIV (2); EbpA (1)	3/90 (3.3)	WZH1 (1); CHG5 (1); CHG19 (1)	5/283 (1.8)	ZHH1 (1); ZHH2 (1); ZHH3 (1); TypeIV (1); EbPigITS7 (1)	4.9 (34/691)	

Prevalence: No. positive/No. sampled (%).

*P*^0^: Shanghai vs Qingdao vs Wenzhou vs Zhuhai; *P*^1^: Males vs Females; *P*^2^: Children vs Adult vs Elderly; *P*^3^: Children vs Adult; *P*^4^: Children vs Elderly; *P*^5^: Adult vs Elderly; *P*^6^: Rural vs Urban.

### 3.2 Genetic characterization of *E. bieneusi* genotypes

Sequencing was conducted on 34 *E. bieneusi*-positive samples. All sequences were successfully accomplished, and 12 representative sequences were acquired. Among these sequences, seven had been previously reported to match the sequences of genotypes CHG3, CHG5, CHG19, EbpA, PigEbITS7, S7, and Type IV. The remaining five sequences were previously unreported and were defined as novel genotypes: SHH1, ZHH1, ZHH2, ZHH3, and WZH1. Genotype SHH1 demonstrates a two - base difference from genotype CHG5. Genotype ZHH1 shows a one - base difference compared to CHG19, genotype ZHH2 has a one-base difference with respect to PL14, genotype ZHH3 presents a one base difference from HNH17, and genotype WZH1 exhibits one base difference from CHG5.

**Figure 2:**
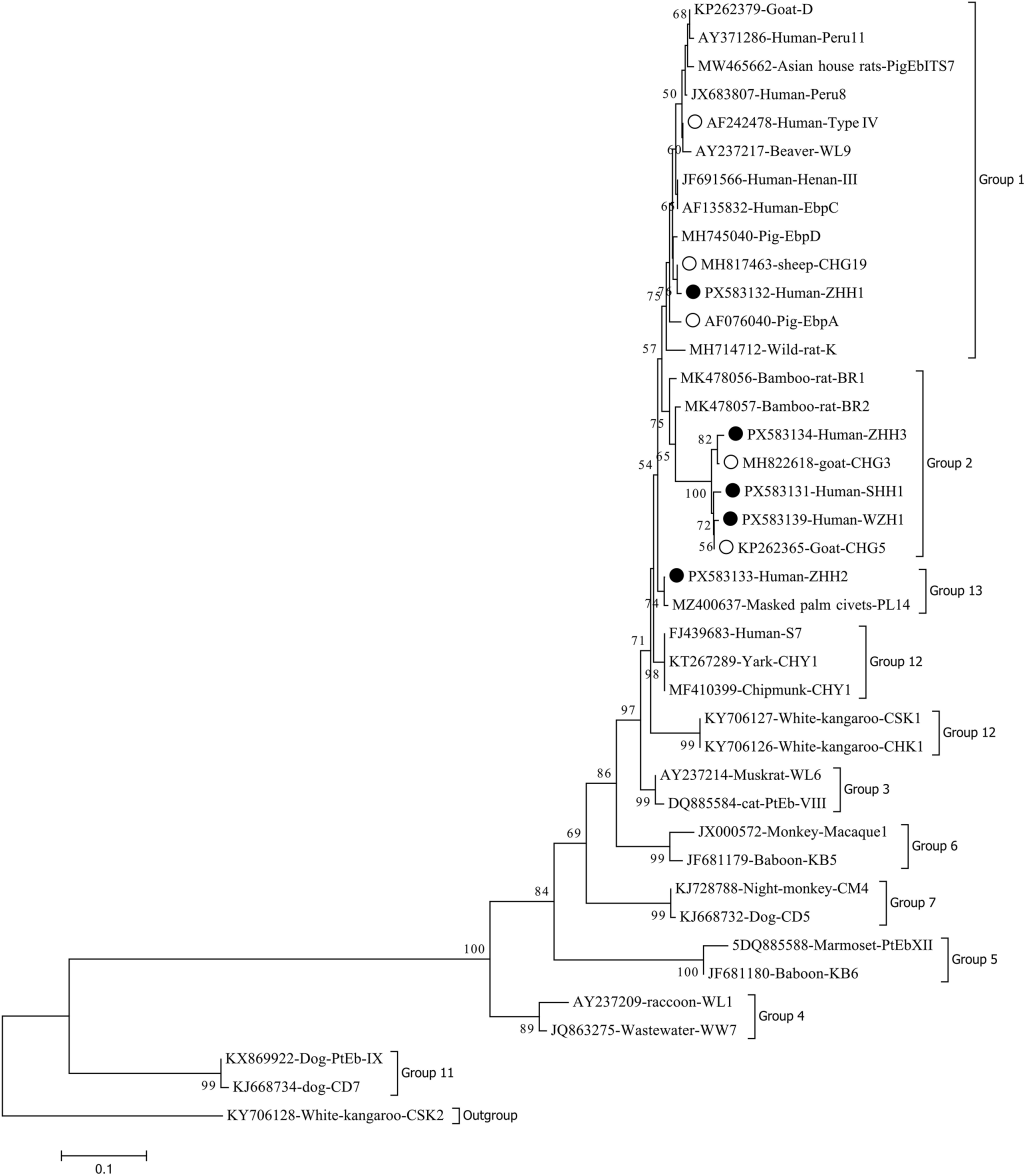
The phylogenetic tree of *E. bieneusi*, constructed base on ITS sequences, implements the neighbor-joining method, rooted in the Kimura 2-parameter model. For validating the tree’s reliability, bootstrap values were acquired through 1,000 iterations. In this tree, the known genotypes and the novel ones identified in the present study are highlighted with hollow and solid circles, respectively.

Phylogenetic analysis revealed that among the 12 genotypes, four known genotypes (EbpA, CHG19, PigEBITS7, and Type IV) and one novel genotype (ZHH1) were categorized into Group 1. Three novel genotypes (WZH1, SHH1, and ZHH3) together with two known genotypes (CHG5 and CHG3) were classified into Group 2, whereas the remaining known genotype S7 and the novel genotype ZHH2 were assigned to Group 12 and Group 13, respectively (**Figure 2**).

### 3.3 Distribution of *E. bieneusi* genotypes

Among the 12 genotypes identified herein, CHG3 constituted the largest proportion (50.0%, 17/34), succeeded by PigEBITS7, Type IV, and CHG19 (each 8.8%, 3/34), and the remaining eight genotypes (each 2.9%, 1/34). The distribution of genotypes exhibits discernible regional disparities: CHG19 is present in both the Shanghai and Wenzhou populations, while TypeIV and PigEBITS7 are found in both the Qingdao and Zhuhai populations. Whereas CHG3, SHH1, and S7 are solely detected in the Shanghai populations; EbPigITS7 and EbpA are exclusively found in the Qingdao populations; WZH1 and CHG5 are only present in the Wenzhou populations; and ZHH1, ZHH2, and ZHH3 are solely detected in the Zhuhai populations (**Table 2**). The distribution of genotypes also exhibits variations among different age groups: children demonstrate a higher degree of diversity, characterized by the presence of nine genotypes. In contrast, adults harbor four genotypes, and the elderly carry five genotypes. Likewise, rural residents possess 10 genotypes, whereas urban populations exhibit six. Significantly, males and females have comparable quantities of genotypes, with eight and nine types respectively (**Table 2**).

## 4 Discussion

*E. bieneusi* infection in humans has been extensively documented globally. Nevertheless, in China, molecular investigations of *E. bieneusi* in human populations have been conducted in only 12 provinces thus far (**Table 1**). Most of these reports focused on *E. bieneusi* infection in HIV-infected populations and children. In the current study focusing on the diarrheal population, the overall positive rate of *E. bieneusi* was 4.9%. Significant differences were detected in the positive rates of *E. bieneusi* among populations from different geographical regions. The positive rate was the highest in Shanghai, reaching 14.3%, which was eight times that in Zhuhai (1.8%). This may be attributed to the relatively high population density in Shanghai. Meanwhile, in Shanghai, 79% of the participants were from rural areas and 54.4% were children, which may also have contributed to the higher positive rate of *E. bieneusi* observed. The positive rates in Wenzhou and Qingdao were relatively close, at 3.3% and 2.9% respectively, indicating a low prevalence level, as these figures were lower than the global prevalence of *E. bieneusi* infection in humans, which was 6.6%, as well as that in China (5.8%) (Wang et al., 2024; Qiu et al., 2019). Overall, there were obvious geographical disparities in the positive rate of *E. bieneusi* among the Chinese population. For instance, two studies in Hubei Province showed significantly low infection rates of 0.2% and 5.0% respectively (Zang et al., 2021; Wang et al., 2017); research in Henan Province also revealed relatively low infection rates of 1.2% and 5.0% (Yu et al., 2019; Wang et al., 2013b); while a study on diarrheal children in Jilin Province presented a relatively high infection rate of 22.5% (Zhang et al., 2011). Among the provinces covered in this study, a previous report from Shandong Province indicated an infection rate of 11.4%, but the sample size was only 44 (Zang et al., 2021). In three investigations carried out in Shanghai, the infection rate ranged from 4.2% to 13.5% (Liu et al., 2014; Wang et al., 2013a; Jiang et al., 2023). Two studies from Zhejiang Province reported different infection rates of 1.6% and 7.2%, with the lower rate observed among healthy farmers and the higher rate among outpatients with diarrhea symptoms (Ye et al., 2025; Liu et al., 2023). Indeed, apart from geographical factors, the infection rate of *E. bieneusi* may be affected by diverse aspects of the research subjects. These encompass the size of the studied population, the disease history of the population, and the immune status of the individuals within that population.

This study finds children have the highest *E. bieneusi* positive rate, followed by the elderly, and adults have the lowest. This is consistent with previous research, which demonstrates that the proportion of microsporidia-positive children (18.8%) is significantly higher than that of adults (10.2%) (Lobo et al., 2012). Children are more susceptible, possibly due to their underdeveloped immune systems and frequent exposure to contaminated environments, such as having poor health habits and lacking proper hygiene awareness after playing games or coming into contact with animals. The higher rate among the elderly may be due to age-related immune decline and comorbidities. Adults, with stronger immune defenses, have the lowest rate (Han et al., 2021). Furthermore, there are significant disparities between the rural and urban populations. Rural residents have a higher positive rate, which could be related to differences in living conditions, hygiene practices, and access to clean water and sanitation. Poor sanitation in rural areas may facilitate transmission through contaminated food or water. Previous research shows elevated infection rates among village-dwelling populations, e.g., rural residents in Myanmar had infection rates up to 8.7% (Shen et al., 2020). Although there’s no conclusive evidence that gender affects *E. bieneusi* infection rates, this study finds a higher prevalence in women than men. In fact, in rural areas, women engage in agricultural work or household chores, and in urban areas, they have closer contact with pets. Therefore, this might be the possible reason why the positive rate of *E. bieneusi* in female patients is higher than that in male patients. In conclusion, the study data further confirm that young children and rural populations are more susceptible to *E. bieneusi* infection. Therefore, some preventive policies should be formulated from the “One Health” perspective. Specifically, it is essential to monitor the infection status of rural children, promote good hygiene practices, and minimize contact with animals. However, the study is hospital-based and cross-sectional, including only diarrheic patients. This limits the comparison of infection rates with non-diarrheic controls. Also, relying on hospital-based data restricts the generalizability of findings to the broader population as it may not represent asymptomatic carriers or mild - symptom individuals who don’t seek medical attention. Future research should include community-based studies with larger and more diverse samples, covering both symptomatic and asymptomatic individuals, to comprehensively understand *E. bieneusi* prevalence and transmission dynamics.

In this study, a total of 12 genotypes were discovered, encompassing seven known genotypes and five novel genotypes. Genotype CHG3 has the highest proportion in this study. It was initially isolated from goats in China and has subsequently been detected in humans in China (Hainan) and Ahvaz, as well as in other animals, including cattle, sheep, geese, and rodents (Shi et al., 2016; Makipour et al., 2025; Zhang et al., 2022; Zhao et al., 2019). Analogously, the genotypes CHG5 and CHG19 were initially recognized in goats and later detected in a variety of other animal hosts, such as pigs, rodents, and geese. Regarding humans, cases associated with CHG5 have been reported in Hainan, China, and no case of CHG19 was documented prior to the present study. Genotype S7, originally detected in a patient in the Netherlands, also has the synonym CHY1 which has been found in yaks, in some pet animals, including chipmunks, rats, and rabbits, as well as in wild rats (ten Hove et al., 2009; Zhao et al., 2020; Deng et al., 2018, 2020). These findings imply that these genotypes, which are exceedingly scarce in the human population, might have been transferred from animals.

Genotypes EbpA, PigEbITS7 and TypeIV are all genotypes that have been widely confirmed to have the potential for zoonotic transmission (Zhang et al., 2021). In addition to human cases being widespread globally, their animal hosts are also very diverse. For example, TypeIV may have over 40 animal hosts (Ruan et al., 2021). Thus, it is difficult to accurately infer the source of human infections. Current data indicate that genotypes EbpA, PigEbITS7, and TypeIV have been identified in various animals in Zhejiang, Shanghai, and Shandong regions (Zhao et al., 2024; Zhang et al., 2025; Ma et al., 2020). More importantly, these genotypes have been detected in urban wastewater treatment plant effluents in Qingdao and Shanghai (Jiang et al., 2024; Li et al., 2012). Previous studies have shown that genotypes EbpA, PigEbITS7, and TypeIV have also been identified in wild rats and shrews in the Wenzhou area (Zhang et al., 2024). This finding implies that these genotypes might have been introduced into the water environment by human activities and subsequently disseminated to other animals or individuals via rodents. Consequently, the One Health approach warrants further consideration to tackle the prevalence of *E. bieneusi*. Measures should be taken from the source to control the infection source and disrupt the transmission pathway.

This research discerned five novel genotypes that exhibited minimal disparities compared to known variants, involving only the exchange of 1 or 2 bases. These genotypes are presumably the outcome of host evolutionary selection. All subsequent variants were initially detected in sporadic cases. For example, BEB6 was first identified in an individual human case in Shanghai, yet subsequently confirmed as the predominant genotype in cattle, sheep, and deer (Wang et al., 2013a; Zhao et al., 2017). Although these novel variants have only resulted in isolated human cases thus far, it is plausible to posit that as research progresses, they may be discovered to have a wider spectrum of hosts, or additional cases may surface. This assertion is supported by phylogenetic analysis, which confirms the hypothesis that all variants are closely related to animal-derived genotypes, with 80% (4 out of 5) originating from Groups 1 and 2. Nevertheless, conclusive determinations necessitate further inquiry, especially through the tracing of animals in close proximity to affected cases.

## 5 Conclusion

Our present study demonstrated the occurrence of *E. bieneusi* among diarrhea patients in four coastal cities of China, as well as the extensive genetic diversity of the genotype constitution of *E. bieneusi*. The presence of all the seven known genotypes (CHG3, CHG19, EbPigITS7, TypeIV, CHG5, EbpA, and S7) identified here was reported in animals previously, which suggests zoonotic transmission. Moreover, the novel genotypes have enriched genetic diversity research. This study provides important evidence for understanding the epidemiological characteristics and genetic diversity of *E. bieneusi* in China, highlighting the need to strengthen monitoring and prevention to curb transmission.

## Data Availability

The datasets presented in this study can be found in online repositories. The names of the repository/repositories and accession number(s) can be found in the article/supplementary material.
